# Allergic conditions and risk of hematological malignancies in adults: a cohort study

**DOI:** 10.1186/1471-2458-4-51

**Published:** 2004-11-04

**Authors:** Karin C Söderberg, Lars Hagmar, Judith Schwartzbaum, Maria Feychting

**Affiliations:** 1 The Institute of Environmental Medicine, Karolinska Institutet, Box 210, S-171 77 Stockholm, Sweden; 2 Department of Occupational and Environmental Medicine, Lund University Hospital, S-221 85 Lund, Sweden; 3 Division of Epidemiology and Biometrics, School of Public Health, Ohio State University, Columbus, OH 43210, USA

## Abstract

**Background:**

Two contradictory hypotheses have been proposed to explain the relationship between allergic conditions and malignancies, the immune surveillance hypothesis and the antigenic stimulation hypothesis. The former advocates that allergic conditions may be protective against development of cancer, whereas the latter proposes an increased risk. This relationship has been studied in several case-control studies, but only in a few cohort studies.

**Methods:**

The association between allergic conditions and risk of developing leukemia, Hodgkin's disease, non-Hodgkin's lymphoma and myeloma was investigated in a cohort of 16,539 Swedish twins born 1886–1925. Prospectively collected, self-reported information about allergic conditions such as asthma, hay fever or eczema was obtained through questionnaires administered in 1967. The cohort was followed 1969–99 and cancer incidence was ascertained from the Swedish Cancer Registry.

**Results:**

Hives and asthma tended to increase the risk of leukemia (relative risk [RR] = 2.1, 95% Confidence Interval [CI] 1.0–4.5 and RR = 1.6, 95% CI 0.8–3.5, respectively). There was also an indication of an increased risk of non-Hodgkin's lymphoma associated with eczema during childhood (RR = 2.3, 95% CI 1.0–5.3).

**Conclusion:**

In contrast to most previous studies, our results do not indicate a protective effect of allergic conditions on the risk of developing hematological malignancies. Rather, they suggest that allergic conditions might increase the risk of some hematological malignancies.

## Background

An association between allergic conditions and cancer risk has been the subject of several epidemiological studies. Both positive and negative associations have been observed and two hypotheses have been formulated to explain such relationships. First, the immune surveillance hypothesis, which proposes that allergic conditions may lead to a decreased risk of malignancy by enhancing the ability of the immune system to detect and eliminate malignant cells [1]. Evidence from some previous studies of hematological malignancies in relation to allergic conditions primarily supports this hypothesis [2-4]. Second, the antigenic stimulation hypothesis states that immune-stimulating conditions lead to an increased risk of malignancy, which for hematological malignancies is supported by some studies [5,6]. This would be caused by a mechanism where the chronic stimulation induced by the activated cells of the immune system eventually lead to randomly occurring pro-oncogenic mutations in actively dividing cells. There are also a number of studies where no associations between different allergic conditions and hematological malignancies were found, or where inconsistent results were obtained. It is plausible that the association between allergic conditions and cancer risk is complex and that the risk of developing cancer could depend on the specific malignancy and also could be influenced by the type of allergic condition.

Most previous studies are retrospective case-control studies. Recall bias may have influenced the results of retrospective case-control studies that have asked for past allergic conditions after diagnosis of malignancy [2-5,7,8], a problem that partly remains after confirmation of the information on medical history in medical records [9-11] and also when a combination of self-reported information and information from hospital notes and general practioner notes was used [12]. There are only a few cohort studies that have investigated the relationship between allergic conditions and hematological malignancies [6,13-19]. Four of the studies primarily lend support to the antigenic stimulation hypothesis for hematological malignancies, as increased risks, or tendencies toward increased risks, were found for a history of allergy [6,15,18,19], whereas two of them support the immune surveillance hypothesis by showing decreased risks [16,17]. Four of the studies concerned asthma only [13,14,16,17].

The purpose of the present study was to investigate the influence of allergic conditions on the risk of developing leukemia, malignant lymphoma and myeloma in a well-established cohort of Swedish twins, and to see whether the results support the immune surveillance hypothesis or the antigenic stimulation hypothesis. An important strength of this study is that information about allergic conditions and confounding factors has been collected prospectively, and therefore the exposure is not subject to differential misclassification.

## Methods

The study base consists of a cohort of twins followed by the Swedish Twin Registry, which was established in 1961 when questionnaires were sent to all 25,778 individuals in a same-sexed twin pair who were born between 1886 and 1925 and were both living in Sweden in 1961. A response from both twins in a twin pair was required for inclusion in the Swedish Twin Registry (21,870 individuals) [20]. After 1961 they were followed-up as individuals, irrespective of their twin-sibling's status. Out of the responders to the 1961 questionnaire, a new questionnaire was sent to 21,863 eligible subjects in 1963 (response-rate 85.1 %), and in 1967 an additional questionnaire was sent to 20,576 eligible individuals (response rate 81.5 %).

The twin cohort was used as a population-based cohort without considering twin status. The present study includes the 16,539 individuals (7,167 men and 9,372 women), who responded to the questionnaire mailed in 1967, and who were still alive and not previously diagnosed with a hematological malignancy January 1, 1969. The median follow-up time was 23 years for men and 27 years for women. The median age at baseline was 56 years (10^th ^and 90^th ^percentiles; 46 and 71 years, respectively). The cohort was followed from January 1, 1969 until diagnosis of a hematological malignancy, death or end of the study (December 31, 1999), whichever came first. Cancer incidence and date of death were ascertained by record linkage to the Swedish Cancer Registry and the Swedish Cause of Death Registry, respectively. In the Swedish Cancer Registry, leukemia is coded according to ICD-8 during the investigated period, while all other malignancies are coded according to ICD-7. We identified 324 subjects with hematological malignancies; 10 cases of HD (ICD-7 201), 112 cases of NHL (ICD-7 200.0–200.3, 202.0–202.4), 75 cases of myeloma (ICD-7 203) and 134 cases of leukemia (ICD-8 204.0–207.9). The leukemias consist of 3 cases of ALL, 67 cases of CLL, 31 cases of AML, 7 cases of CML and 26 cases of unspecified leukemia.

### Exposure assessment

Assessment of exposure is based primarily on the 1967 questionnaire, in which questions were asked about the allergic conditions asthma, hay fever, eczema and hives, considering both present and past conditions. The questions were posed as "Have you ever had asthma? (No/Yes), "Have you ever had hay fever, rose fever or allergic rhinitis (characterized by running nose, watery and itching eyes when you do not have a cold)?" (No/Yes), "Did you have eczema when you were a baby?" (No/Yes), "Did you at times later in life have eczema-like skin conditions?" (No/Yes), Do you know the name of the skin lesion you have or have had? (No/Yes, psoriasis/Yes, hives (urticaria) allergic rash/Yes, contact eczema/Yes, eczema in knee or elbow fold/Yes, allergic eczema/Yes, others: specify name). There were no questions about dates when symptoms first started or ended, and no information about treatments used. In the questionnaire from 1963 responses to two questions about asthma and eczema were included. The subjects could mark if they had had asthma or eczema from a list of 17 diseases or the alternative that they hadn't had any of the given diseases. The answer from 1963 was used only if a subject had failed to answer the corresponding question in the 1967 questionnaire. Each medical condition was analyzed separately. In addition we combined the different conditions in order to achieve larger numbers of individuals and thereby obtain more precise results. First, a variable for eczema was created, requiring at least one positive answer for childhood eczema or allergic eczema. Then, a general group of allergic conditions was created, combining the positive answers for eczemas with positive answers on the questions of ever having had asthma or hay fever.

### Confounders and effect modifiers

All analyses were adjusted for age at enrolment and sex. We have controlled for confounding from alcohol consumption (g/month), level of education, and smoking habits (non-smokers, former smokers, current smokers). Adjustment for these factors did not affect the risk estimates in the majority of analyses, and changed the magnitude of the effect at the most 6% in a few instances. Therefore, the presented results are only adjusted for age and sex.

### Statistical methods

We estimated the RR and its 95% CI of each hematological malignancy through Cox's Proportional Hazards Model, (SAS program PHREG, SAS Institute, Cary, North Carolina). To ensure that confidence intervals were not erroneously narrowed due to dependencies within twin pairs we performed analyses that adjusted variance estimates for correlated outcomes. We accomplished this through the use of a SAS macro that stems from the same theoretical background [21-24] and yields the same results as the published Fortran program of D.Y. Lin [24]. In simple terms, variance estimates are increased in magnitude proportional to the degree of extra correlation within twin pairs. Thus, adjusted confidence intervals are more conservative than unadjusted. If correlations within twin pairs are not different from what is observed between unrelated individuals in the cohort with respect to cancer risk, adjusted and unadjusted variance estimates are identical. Relative risk estimates are not altered by this procedure.

## Results

Our results showed either increased risks or risks close to unity for hematological malignancies following allergic conditions. For leukemia, we found an increased risk associated with hives, and an indication of elevated risk associated with asthma, although with wide confidence intervals (Table 2). For leukemia, excluding CLL, the increased RR associated with hives was further elevated.

**Table 2:** Age- and sex-adjusted relative risks for leukemia among subjects with allergic conditions.

	**Leukemia**				**Leukemia, excluding**				**CLL**			
		
***Exposure***	*N*_*e*_	*N*_*o*_	*RR*	*95% CI*	*N*_*e*_	*N*_*o*_	*RR*	*95% CI*	*N*_*e*_	*N*_*o*_	*RR*	*95% CI*
		
**Asthma, hay fever or hives**	31	94	1.4	(0.9–2.1)	21	43	2.0	(1.2–3.4)	10	51	0.8	(0.4–1.6)
**Hay fever**	21	105	1.1	(0.7–1.8)	14	50	1.5	(0.8–2.8)	7	55	0.7	(0.3–1.6)
**Asthma**	7	126	1.6	(0.8–3.5)	3	64	1.3	(0.4–4.2)	4	62	1.9	(0.7–5.3)
**Hives**	7	120	2.1	(1.0–4.5)	6	58	3.6	(1.6–8.5)	1	62	0.6	(0.1–4.3)
Eczema*	8	115	0.9	(0.5–1.9)	3	59	0.7	(0.2–2.2)	5	56	1.2	(0.5–3.1)
Allergic conditions**	30	87	1.1	(0.8–1.7)	18	42	1.4	(0.8–2.4)	12	45	0.9	(0.5–1.7)

N_e _= No. of exposed cases, N_o _= No. of unexposed cases.

* At least one positive answer for eczema during childhood or allergic eczema.

** At least one positive answer for asthma, hay fever, eczema during childhood or allergic eczema.

The risk estimates for myeloma were generally close to or below unity (Table 3). The number of cases with HD was small, with few exposed cases in all analyses making results difficult to interpret (data not shown). For NHL, the risk associated with having had eczema during childhood was increased.

**Table 3:** Age- and sex-adjusted relative risks for myeloma and non-Hodgkin's lymphoma among subjects with allergic conditions.

	**Myeloma**				**non-Hodgkin's**			
	
***Exposure***	*N*_*e*_	*N*_*o*_	*RR*	*95% CI*	*N*_*e*_	*N*_*o*_	*RR*	95% CI
	
**Asthma, hay fever or hives**	10	58	0.7	(0.4–1.4)	22	83	1.1	(0.7–1.8)
**Hay fever**	10	62	0.9	(0.5–1.7)	21	87	1.3	(0.8–2.2)
**Asthma**	0	75		-	0	11		-
**Hives**	1	67	0.5	(0.1–3.9)	1	10	0.4	(0.0–2.6)
Eczema*	3	67	0.6	(0.2–2.0)	8	87	1.3	(0.6–2.6)
**Eczema during childhood**	1	69	0.5	(0.1–3.7)	6	89	2.3	(1.0–5.3)
Allergic conditions**	12	55	0.7	(0.4–1.4)	27	69	1.3	(0.8–2.0)

N_e _= No. of exposed cases, N_o _= No. of unexposed cases.

* At least one positive answer for eczema during childhood or allergic eczema.

** At least one positive answer for asthma, hay fever, eczema during childhood or allergic eczema.

## Discussion

Our results suggested that allergic conditions are risk factors for hematological malignancies, and gave support to the antigenic stimulation hypothesis. Thus, the results are in concordance with most previous cohort studies [6,15,18,19].

A major strength of the present cohort study is that information about allergic conditions and confounding factors has been collected prospectively. Therefore the exposure is not subject to differential misclassification, which is in contrast to retrospective case-control studies where recall bias may be a problem [2,3,9] and where separating the effects of prior allergic conditions from the effect of malignancy *per se *on the immune system may be difficult. This study has focused specifically on how allergy influences the risk of developing hematological malignancies.

Another strength is that the study is based on the Swedish Twin Registry, which is a unique resource allowing for an unusually long period of follow-up. In our study, 31 years of follow-up was possible. The cohort has been followed continuously in the Population Registry and the Cause of Death Registry during the study period, and therefore loss to follow-up is unlikely to be a problem. The Swedish Twin Registry is considered a study base representative of the general population of Sweden and has been used in many epidemiological studies [25-27]. Another strength is the completeness of the Swedish Cancer Registry, to which it is required by law to report all incident cancer cases in Sweden. New cases of cancer are reported by physicians in hospitals and other establishments as well as by pathologists. The two independent notifications systems ensure a high coverage. In addition, we could adjust for more confounding factors than in previous cohort studies [6,13-19].

One limitation of the study is the small number of exposed cases, and therefore random variation cannot be excluded as an explanation for our findings and for the same reason no stratification for calendar time was performed. Another limitation in the study is that there may be non-differential misclassification of the malignancies. The study period covers 31 years, and during this time diagnostic practices may have changed. In particular, some cases previously diagnosed as HD are now likely to be classified as NHL [28]. This type of error would bias the effect estimates towards unity.

Differential misclassification of exposure is unlikely as there is no reason to believe that reporting exposure should differ between subjects subsequently (years later) diagnosed with a cancer, and those who are not. Non-differential misclassification of exposure is likely to affect the results, but cannot explain increased risks since it would dilute the effect estimates towards unity. The allergic conditions are self-reported and not diagnosed by a physician. However, these self-reported conditions have been used in an earlier study of brain tumors, where some support for the postulated hypothesis that allergic conditions are associated with a decreased risk of developing glioma was found [29]. Also, the validity of the allergic conditions has been investigated in a group of subjects from the Swedish Twin Registry [30]. In general, a good agreement was found between the self-reported conditions and an allergologist's diagnosis. Follow-up starts in 1969 and continues until the end of 1999. During 31 years it is possible to develop an allergic condition, but these individuals will still be considered as unexposed members of the cohort. However, the youngest individuals in our cohort were 42 years old when the questionnaire was sent out, which means that this bias have not at all affected childhood eczema, and hay fever and allergic asthma only to a small extent, as these conditions usually present earlier in life.

We found an increased risk of NHL among individuals with eczema during childhood. In the literature, there are only few studies concerning an association between eczema and NHL. In one study, a history of eczema was associated with an increased risk of NHL [10]. In several studies, elevated risks for different hematological malignancies among persons with eczema have been found, e.g. [7,11]. On the other hand, eczema has also been observed to decrease the risk of NHL in two studies [3,9]. Comparisons between these other studies and our study are difficult, however, since the other studies have investigated general eczema while we have focused on eczema of allergic origin (i.e. allergic eczema and eczema during childhood). When using the general definition for eczema many non-allergic forms will be included and while these may influence the risk of developing malignancies, the mechanisms involved are probably different from the ones active in allergic conditions. Thus, these eczemas are not included in the present study.

In our material subjects with hives showed an increased risk of leukemia, especially after exclusion of CLL. Several other studies have also found an increased risk of AML [12] and other hematological malignancies associated with hives [6,9,18]. In contrast, some other studies did not show this association [8]. A number of studies have found a protective effect of asthma on the risk of developing lymphatic leukemia and leukemia, respectively [16,17]. This relationship between asthma and leukemia was not confirmed in our study. If anything, our results support the antigenic stimulation hypothesis. In a recent cohort study, an increased risk of leukemia was indicated [19]. On the other hand, most studies of hematological malignancies in relation to a history of asthma have shown risks close to unity [8,9].

Clearly, these conflicting results indicate that this area needs to be investigated further. Allergic conditions, like asthma and hay fever, are increasing and it is of great importance to clarify if and how they are connected to hematological malignancies. The contradictory findings may have many explanations, e.g. that different immunological mechanisms may be involved in different types of asthma, that the pathogenesis is likely to be different even in seemingly similar hematological malignancies, and that new forms of pharmacological therapy may influence not only the outcome of asthma but also the risk of developing cancer. To solve these problems, large prospective epidemiological studies on individuals with clinically strictly defined allergic conditions, including data on pharmacological treatment and severity of disease, need to be combined with information about morphologically defined hematological malignancies, including subtyping with techniques from modern molecular biology.

## Conclusions

In summary, findings from our cohort study suggest that chronic antigenic stimulation from allergic conditions might increase the risk of some hematological malignancies.

## Abbreviations

ALL = Acute lymphoblastic leukemia; AML = Acute myeloid leukemia; CLL = Chronic lymphocytic leukemia; CML = Chronic myeloid leukemia; HD = Hodgkin's disease; NHL = Non-Hodgkin's lymphoma; RR = Relative risk; CI = Confidence Interval.

## Competing interests

The author(s) declare that they have no competing interests.

## Authors' contributions

KCS has been the principal investigator, contributed to the planning of the study, performed the statistical analysis and drafted and coordinated the writing of the manuscript. LH participated in the planning of the study and the writing of the manuscript. JS contributed to the writing of the manuscript. MF carried out the study design and contributed to the writing of the manuscript. All authors contributed to the interpretation of results, have read and approved the final manuscript.

**Table 1:** Self-reported allergic conditions among 16,539 subjects.

**Self-reported allergic conditions**	**Number of respondents**	**Number reporting condition**	**% reporting condition**
**Asthma, Hay fever or Hives**	15,168	3,022	19.9
**Hay fever**	15,546	2,428	15.6
**Asthma**	16,376	604	3.7
**Hives**	15,379	430	2.8
Eczema*	14,803	1,033	7.0
**Eczema during childhood**	14,816	400	2.7
Allergic conditions**	14,294	3,430	24.0

* At least one positive answer for eczema during childhood or allergic eczema.

** At least one positive answer for asthma, hay fever, eczema during childhood or allergic eczema.

## Pre-publication history

The pre-publication history for this paper can be accessed here:


